# Experiences That Matter: Unraveling the Link Between Extracurricular Activities and Emotional and Social Competencies

**DOI:** 10.3389/fpsyg.2021.659526

**Published:** 2021-08-19

**Authors:** Laura Cortellazzo, Sara Bonesso, Fabrizio Gerli, Claudio Pizzi

**Affiliations:** ^1^Department of Management, Ca'Foscari University, Venice, Italy; ^2^Department of Economics, Ca'Foscari University, Venice, Italy

**Keywords:** emotional and social competencies, experiential learning, extracurricular activities, PLS path modeling, emotional intelligence

## Abstract

Emotional and social competencies have been shown to be extremely desirable in young people for their successful entry into the labor market. Their development has been studied primarily as a result of formal training in the educational and work domains, whereas relatively little is known about the role played by extracurricular activities in promoting these types of competencies. Non-working personal experiences are often used as proxies to assess the emotional and social competencies of candidates in recruitment and selection phases. However, this inference is not based on clear scientific evidence. Drawing on experiential learning theory, this study investigated empirically the relationship between a range of extracurricular activities (volunteering, cultural activities, experience abroad, sport) and the competency portfolio of graduates. Data were collected from a sample of 324 graduates through a structured survey and a multi-rater assessment of their emotional and social competencies. The results of the Partial Least Square-Path Modeling in general provide support for the positive association between experiential extracurricular activities and emotional and social competencies, although not all relationships are supported. The present study contributes to advance in the understanding of the determinants of emotional and social competencies by examining their relationship with a broad range of extracurricular activities. Moreover, it discusses implications for higher education and human resource management.

## Introduction

The current economic environment, characterized by increasing competition, flexibility, and continuous rapid change, has led companies to look for new employees with personal characteristics that go beyond their mere technical ability. Nowadays, people who want to enter the labor market are required to show a set socio-emotional competencies (Garcia-Arracil and Van der Velden, 2008) that enable them to pursue effectiveness (Brown et al., 2003; Emmerling and Cherniss, 2003; Williams, 2008; Beigi and Shirmohammadi, 2011; Emmerling and Boyatzis, 2012; Zhang and Fan, 2013). Adopting a behavioral approach to emotional and social competencies (ESCs; Boyatzis, 2016), we define them as “related but different sets of behavior organized around an underlying construct, which we call the ‘intent” (Boyatzis, 2009, p. 750) that differentiate effective job performers (Dulewicz and Herbert, 1999; Salas Velasco, 2014). Indeed, previous studies showed that ESCs contribute to personal and professional success (Sigmar et al., 2012; Boyatzis et al., 2017), and employability (Hogan et al., 2013). Currently, they are the most in-demand competencies in the labor market (Azevedo et al., 2012; LinkedIn, 2019). However, the evidence of a competency gap, especially in the young workforce, is still widely attested across countries (Jackson, 2009; QS Intelligence Unit, 2019). In order to reduce this gap and help individuals be more attractive when entering the labor market, understanding individual differences in the development of these competencies has become paramount (Leimbach and Maringka, 2010).

Scholars maintain that the development of these competencies requires an approach that is different from traditional methods based on passive accumulation of knowledge that have traditionally been offered by higher education institutions (Garcia-Arracil and Van der Velden, 2008). The development of ESCs requires an approach focused on active learning, stimulation of relationships and cooperation (Garcia-Arracil and Van der Velden, 2008), in which experience plays a critical role (Kolb, 1984; Ng et al., 2009). Following this approach, some institutions have started to supplement their traditional educational activity by integrating experiential learning exercises into academic courses (Boyatzis et al., 2002; Hoover et al., 2010). However, the vast majority of people that do not have access to this type of training activities are supposed to rely on their own personal experiences. While scholars have mainly investigated whether ESCs can be developed through training and development programs (Cherniss, 2000; McEnrue and Groves, 2006; Miao et al., 2020), the literature has neglected that the acquisition of these abilities may also be pursued through other experiences, such as responsibilities held in student organizations or clubs, internships, or summer jobs (Salas Velasco, 2014). According to Rubin et al. (2002, p.441), “one intuitive notion is that extracurricular activities are a place where students look to utilize, and perhaps refine and develop, their interpersonal skills.” Indeed, practitioners often use extracurricular activities (ECAs) as proxies to assess personal abilities in phases of recruitment and selection, inferring the acquisition of some ESCs (Cole et al., 2007). Recruiters ascribe individuals' skills from the candidates' ECAs according to the type of activity performed (Hutchinson, 1984; Newanick and Clark, 2002; Brown and Hesketh, 2004; Rivera, 2011). However, this inference seems to be guided by “good instinct” (Graham-Leviss, 2012), rather than by scientific evidence, as research has devoted marginal attention to the role of ECAs (Moore, 2013).

This paper contributes to the filling of this void by analyzing the relationship between graduates' extracurricular experiences and their ESC portfolio. Our work adds to the literature by advancing the understanding of the determinants of ESC. Specifically, we shed light on the role of different ECAs (volunteering, cultural activities, experience abroad, sport) in promoting specific sets of ESCs (self-awareness, self-management, social awareness, relationship management, cognitive competencies), and discuss the implication of these relationships for higher education and human resource management.

## Theoretical Background

### The Development of ESCs Through Experiential Learning

Behavioral competencies are defined as “related but different sets of behavior organized around an underlying construct, which we call the ‘intent” (Boyatzis, 2009, p. 750). Emotional and social intelligence competencies are classified in three main groups (Boyatzis, 2009): (i) emotional competencies that encompass both the ability to recognize one's own emotions (emotional awareness) and to manage those emotions even in critical circumstances (self-management); (ii) social competencies that include both the ability to recognize others' emotions (social awareness) and to manage emotions in interpersonal relationships (relationship management); (iii) cognitive competencies as the ability to analyze information and situations (Boyatzis et al., 2019).

Adopting a behavioral perspective (Boyatzis, 2016), acquiring or improving ESCs ultimately requires a change in one's common behavior. Decades of research in different fields, such as psychotherapy, training programs, and education, have shown that people can actually change their behavior (Cherniss and Goleman, 2001; Boyatzis, 2008b). However, scholars also claim that non-traditional methods in which the person is involved in an emotional and experiential context need to be adopted in order to develop ESCs (Kremer and McGuinness, 1998; Dwyer, 2001). By involving participants in a process of reflection, interactive engagement, and practice, experiential learning techniques stimulate the cognitive, behavioral, and emotional dimensions of learning and behavioral change that are necessary to acquire ESCs (Hoover et al., 2010). Experiential learning conceives learning as a holistic process in which the person is called upon to think, feel, perceive and behave in the interaction with the environment (Kolb, 1984; Ng et al., 2009). The experiential learning model portrays two dialectically related modes of grasping experience—Concrete Experience and Abstract Conceptualization—and two dialectically related modes of transforming experience—Reflective Observation and Active Experimentation (Kolb and Kolb, 2005). According to experiential learning theory, although individuals may be characterized by a preferred mode, the ideal learning cycle is a recursive process in which all four modes are adopted (Kolb and Kolb, 2005). Concrete experiences represent the basis for reflection and observation. According to their perceptions, individuals attach meanings to their experiences, creating frameworks of knowing (Rigg, 2008). The meaning created transforms the action patterns, leading people to experiment with new behaviors (Yeo and Marquardt, 2015). Experiential learning theory is based on key pillars that challenge the traditional idea that learning is achieved through transmission of knowledge, and claims that learning is a process of creating knowledge through the synergetic transactions between the person and the environment. ECAs represent a set for the application of experiential learning as they expose students to concrete experiences in different environments that challenge their perceptions and behaviors. Consistent with experiential leaning theory, this allows students to re-examine, test and integrate their beliefs and behavior. Through active involvement in ECAs (concrete experience), students have the opportunity to observe and reflect on their current beliefs and behaviors and to identify, model and mirror appropriate behaviors (reflective observation). The critical thinking that emerges from reflection (abstract conceptualization) can enhance the practice of new or more suitable behaviors (active experimentation). ECAs imply the involvement in a structured or semi-structured organization, in which objectives that can be pursued alone or in a group are usually defined (Mahoney et al., 2003). Compared to leisure activities, which may be undertaken just for fun, the engagement in a structured activity that entails the activation of conscious behaviors in addressing objectives is more likely to drive the individual through the four stages of the learning cycle. For instance, in extracurricular activities such as an experience abroad, students have the opportunity to reflect upon themselves and to analyze critically their behavior compared to international colleagues. In music and sports, which often involve formal or informal feedback by experts and audiences (Alessandri et al., 2020), individuals acquire information about their behavior and can use it to improve and better address their objectives. Nevertheless, compared to forced or simulated experiences, prior studies maintain that extracurricular experiences represent a more reflective and revelatory ambience, which facilitates the transformation of behavioral patterns (Nair, 2011).

### Extra-Curricular Activities and Emotional and Social Competencies

Previous research supports a positive effect of participating in ECAs on people development, especially at a young age (Rubin et al., 2002; Fredricks and Eccles, 2006). Prior studies showed a positive association with academic outcomes (e.g., Cooper et al., 1999; Eccles and Barber, 1999), lower depression (Mahoney et al., 2002), and higher self-esteem (Fredricks and Eccles, 2006). Participating in ECAs provides students the opportunity “to acquire and practice specific social, physical, and intellectual skills that may be useful in a wide variety of settings” (Eccles et al., 2003, p. 866), thus, it equips individuals with those general transferable skills important for labor market outcomes (Salas Velasco, 2014). For instance, among the few studies that investigate the relationship between ECAs and ESCs, Rubin et al. (2002) found a positive relationship between the participation in ECAs and students' communication, initiative, decision-making, and teamwork skills (Rubin et al., 2002). Other scholars have reported significant relationships between participation in ECAs and constructs such as self-concept (Yarworth and Gauthier, 1978; Haensley et al., 1986; Eccles and Barber, 1999). Still others found positive relationships with the general construct of interpersonal competence (Howard, 1986; Fredricks and Eccles, 2006). However, the literature does not provide an extensive investigation of the role of different types of ECAs in enhancing different types of ESCs, and extant empirical research provides inconclusive results (Kim and Bastedo, 2016).

**Figure 1:**
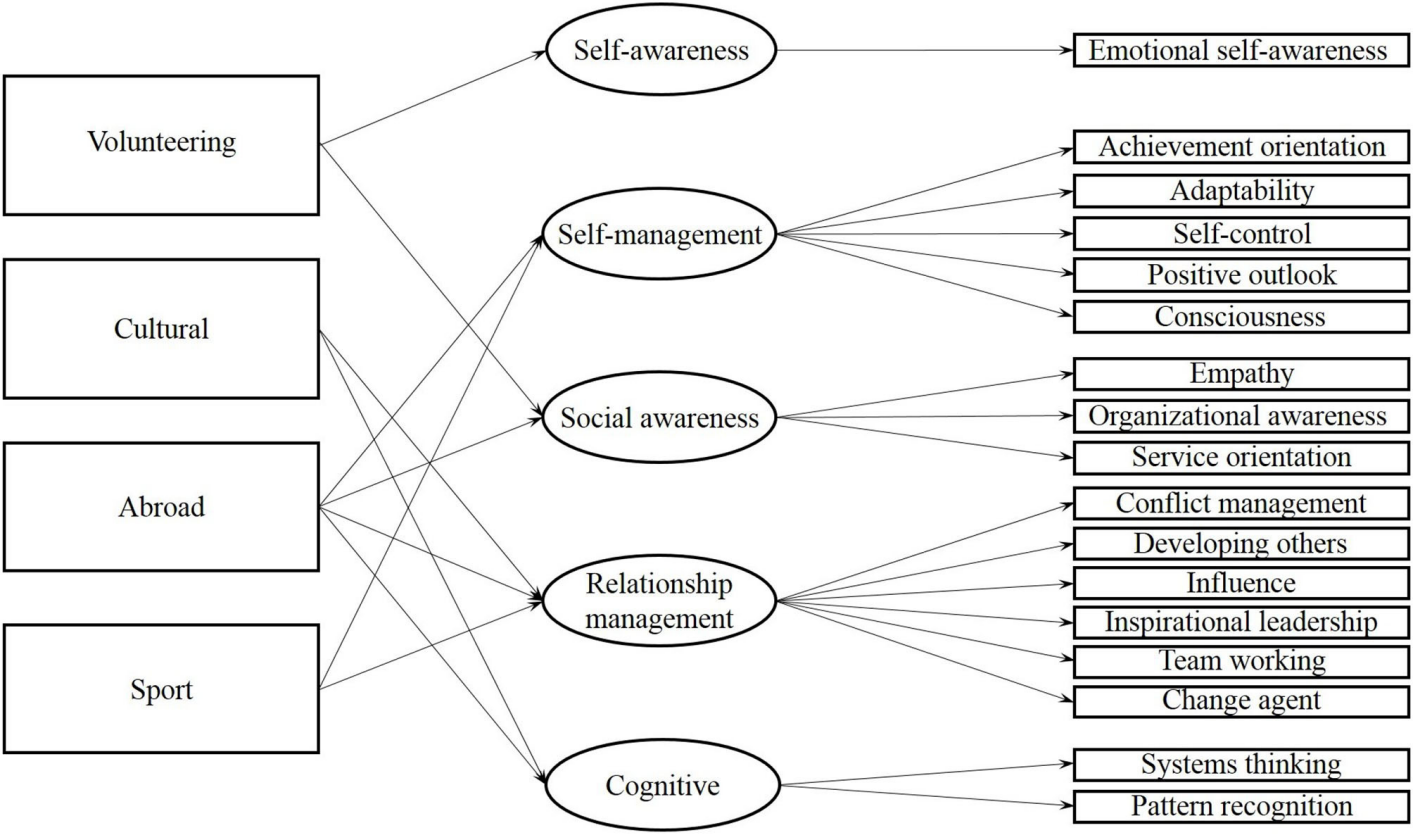
Conceptual model.

In the literature, different types of ECAs are identified: sport (Rubin et al., 2002; Forneris et al., 2015), community service and volunteer work (Ward and Yates, 2012), community fundraising clubs (Forneris et al., 2015), internship and study abroad activities (Ward and Yates, 2012), art clubs, drama clubs and music (Forneris et al., 2015) and on-campus clubs and fraternities/sororities (Forneris et al., 2015). In this study, we focused on four main categories: volunteering, cultural activities, experiences abroad and sport, and we differentiate the relationships of different types of ECAs with multiple groups of ESCs (Figure 1). Specifically, we adopted the conceptual model of Boyatzis and Sala (2004), which distinguish ESCs into five main groups: self-awareness, self-management, social awareness competencies, relationship management and cognitive competencies. Self-awareness concerns the ability to deeply understand oneself and one's emotions, abilities and limits. Self-management relates to the ability to effectively use one's emotions and manage oneself. Social awareness allows an understanding of other people, their emotions, behaviors and points of view, while managing interpersonal relationship and social situations relates to relationship management. Cognitive competencies allow an understanding of complex phenomena and recognizing patterns. A detailed definition of the ESC groups is reported in Table 1.

**Table 1:** Definition of groups and competencies.

**Competency group**	**Definition**	**Competencies included**
Self-awareness	The ability to understand your own emotions and their effects, to know your abilities and limits	Emotional self-awareness
Self-management	The ability to manage and use your own emotions to be more effective	Achievement orientation Adaptability Self-control Positive outlook Consciousness
Social awareness	The ability to understand what people feel, their point of view, cultivating positive relationships	Empathy Organizational awareness Service orientation
Relationship management	The ability to manage emotionally interpersonal relationships, clearly read social situations and relationships, interact without friction	Conflict management Developing others Influence Inspirational leadership Team working Change agent
Cognitive	The ability to understand complex phenomena and recognize the underlying patterns in situations or events	Systems thinking Pattern recognition

Volunteer activity is defined as work done without monetary recompense (Freeman, 1996). Previous studies have focused on volunteering outcomes, such as promoting a sense of democracy (Sǎveanu and Sǎveanu, 2013), and having a positive effect on individual well-being, with positive physical and mental health consequences (Thoits and Hewitt, 2001) leading to greater life satisfaction (Meier and Stutzer, 2008). In recent years, volunteering has gained relevance in the European context as a way to improve employability through skills development (European Commission, 2011). Previous studies have maintained that volunteering is a way to develop an active identity (Sǎveanu and Sǎveanu, 2013) by stimulating the assessment of one's abilities, values, interests and place in the society (Eccles et al., 2003; Brown-Liburd and Porco, 2011), thus influencing one's self-awareness. Moreover, volunteering allows students to change their perception on “the other” (Youniss and Reinders, 2010) and their perspective on people and their problems (Reed, 2001), showing empathic abilities.

Under these considerations, we expect that:

H1: Students who have experienced volunteering more frequently demonstrate higher emotional awareness and social awareness competencies.

Cultural activities are defined as activities related to fine arts such as music, theater and visual arts. In the literature, cultural activities are recognized as improving people's development, especially concerning creative and cognitive competencies (Burton et al., 2000; Snyder et al., 2009). Arts education contributes to spur qualitative judgment and the ability to combine elements without the help of a rule or formula; it may foster the creation of scenarios, and develop a “willingness to imagine possibilities” (Eisner, 1998). According to Jacques (2012), some common techniques used in the theater may help develop the ability to think in a non-linear fashion. Both listening to music and music lessons have been widely related to the enhancement of students' cognitive abilities, even if empirical evidence is based mainly on correlational studies (see Schellenberg et al., 2007 for a review). Previous findings have claimed that listening to music enhances spatial-temporal reasoning (Rauscher et al., 1995; Wilson and Brown, 1997), children's cognitive performance and creativity (Schellenberg and Hallam, 2005; Schellenberg et al., 2007), leads to improved brain efficiency (Thompson et al., 2001; Gupta et al., 2018) and helps students perceive patterns (Magne et al., 2006). Scholars suggests that art activities may also play a role in spurring relationship management competencies. Jacques (2012, p. 247) claims “Theater has a long, well-established system of learning interpersonal skills,” such as a positive influence on listening, giving feedback, collaborating and problem solving (Lesavre, 2012). One of the main outcomes of theater activity refers to relationship building (Huffaker et al., 2003; Dominguez et al., 2007) through collaboration, reciprocity and the development of a sense of group harmony, which is necessary to put a performance on stage. Similarly, Schumacher (2009) investigated the role of music in the improvement of social skills. Previous studies based on laboratory experiments attempted to explain the role of music in creating social bonds by looking at its biological basis (see Chanda and Levitin, 2013). Synchronized activities, such as music, were found to foster feelings of social connection, specifically interpersonal trust and bonding (Chanda and Levitin, 2013).

According to these considerations, we expect that:

H2: Students who have experienced cultural activities more frequently demonstrate higher relationship management and cognitive competencies.

With the growth of an international dimension in both the professional and the academic fields, there is little doubt that experiences abroad can confer benefits for all students (Ungar, 2016). An international experience fits naturally under the concept of experiential learning, as it is a learning experience that is transformed through the active participation of the student through a process that entails observing, discussing and questioning (Sjoberg and Shabalina, 2010) and exposes students to the challenges of living in a different environment (Ng et al., 2009). Scarinci and Pearce (2012) note that experiences abroad can result in a variety of outcomes, such as skill development and cognitive, attitudinal and behavioral learning (Schuster et al., 1998). The cultural immersion that students experience expands their worldview, reduces prejudice and develops cultural sensitivity (DeRicco and Sciarra, 2005; Ishii et al., 2009; Hipolito-Delgado et al., 2011). In order to reduce the effects of cultural shock, students need to understand better the behaviors and perspectives of others and challenge stereotypes. An enhanced social awareness favors the ability to manage effectively one's behavior in adapting to the foreign culture. This is an opportunity to develop an attitude toward change and to be able to face adversities (Sell, 1983). If, on one hand, the removal from their home environment provides students with freedom from familial or cultural constraints and expectations and gives them a stimulus to explore new possibilities and to experiment with different behaviors (Brown, 2009), on the other it may hide unexpected situations that are difficult to manage, such as cultural differences, language difficulties and independence. Studying and working abroad is also a chance to evolve the student's ability to build and manage relationships with people from different backgrounds, thus enhancing their social competencies, which are indeed the main characteristics that employers require from graduates with international experience (Jones, 2013). Lastly, an international experience may favor cognitive competencies, since students have the possibility to evaluate cultural differences, gain holistic insights into a specific social system and analyze and reflect on similarities and differences between cultures.

In respect of experiences abroad, we expect that:

H3: Students who have undertaken experiences abroad for a longer period demonstrate higher self-management, social awareness, relationship management and cognitive competencies.

One of the most widespread extracurricular activities in modern society is sport (Forneris et al., 2015). Moreno-Murcia et al. (2011) summarize some of the benefits recognized by social sciences of participating in sport: it promotes physical development (Malina et al., 2004), self-esteem (Fox and Corbin, 1989), and prevents physical and psychological problems such as obesity (Bar-Or et al., 1998), anxiety and depression (Alfermann and Stoll, 2000; Fox, 2000). Moreover, sport activities represent opportunities to strengthen competencies related to both the self and others. Firstly, sport activities are a way to strengthen the ability to manage oneself. Student athletes commit a great deal of time, energy and emotional involvement to their sports (Sauer et al., 2013) and need dedication and discipline, which implies consciousness and often results in a great sense of achievement orientation. Especially during performances, matches and competitions, students have to manage and control their emotions and reactions. Secondly, sport activities are often related to social management skills, such as leadership (Kniffin et al., 2015), teamwork and relationship management skills (Sauer et al., 2013), particularly in group-based activities. Experience in college athletics is the environment where many managers reported that they had their first opportunity to implement group management tactics (Sauer et al., 2013).

Therefore, our fourth hypothesis states that:

H4: Students who have undertaken sport experiences more frequently demonstrate higher self-management, and relationship management competencies.

## Method

The present study was carried out on a sample of 324 students in Master's degree programs in an Italian setting. The sample consisted of 70% females and 30% males, which is explained by the gender composition of the students enrolled in the university (67.2% female) (MIUR, 2017). Sixty percent of the sample came from an economic–scientific field, with the remaining 40% from the humanistic–linguistic field. Average age was 24.66 years (SD = 2.87).

### Measures

#### Extracurricular Activities

We asked students the intensity with which in the past they had participated in different types of ECA, using a scale from 1 (never) to 5 (more than twice a week). Previous studies suggest that frequent exposure to an activity is needed to experience the positive developmental outcomes associated with participation in that environment (Larson and Verma, 1999; Hansen and Larson, 2007). This is coherent with the theories of ESC learning, according to which the more a behavior is practiced, the more it becomes an automatic response and thus translating into a persistent competency (Goleman et al., 2002; Boyatzis, 2008a; Rock and Ringleb, 2013).

#### Emotional and Social Competencies

The Emotional and Social Competency Inventory in the edition applicable to university students (ESCI-U) was used (Boyatzis and Sala, 2004). Three competencies included in the previous version of the model (ECI—Emotional Competency Inventory) were integrated into the survey: consciousness, service orientation and change agent. Table 1 summarizes the groups of competencies included in the model, which were assessed through 79 behavioral indicators, using a scale from 0 (never demonstrated) to 10 (always demonstrated).

Prior research determined the reliability and validity of the ESCI-U scale as well as of the previous versions of the model, including in cross-cultural contexts (Boyatzis et al., 1999, 2015; Boyatzis and Sala, 2004; Sharma, 2012; Padilla-Meléndez et al., 2014). The model consists of a 360-degree assessment. The use of self-reported data has suffered a number of criticisms concerning that self-ratings are likely to suffer from leniency and social desirability bias (Podsakoff and Organ, 1986). Similar conclusions were drawn from the emotional and social competency literature (Boyatzis et al., 2002; Baumeister, 2005; Clarke, 2010; Taylor, 2010). Several lines of evidence suggest that the ratings given by other people provide a more complete picture of an individual's behavior (Taylor and Bright, 2011), and that a 360-degree assessment excluding self-assessment is less likely to be susceptible to bias (Bernardin and Tyler, 2001). Moreover, scholars in the emotional and social competency field generally report lower reliability indices for self-assessment measures compared to external assessment (i.e., Boyatzis et al., 2015). Thus, following previous studies (i.e., Hopkins and Bilimoria, 2008; Boyatzis and Ratti, 2009; Dragoni et al., 2009) we conducted the analysis using external evaluations only and calculated the average of the 360-degree assessment across all rating sources for each competency (Hagan et al., 2006). The literature highlighted that aggregated scores of external raters represent the most valid predictor of performance (Atkins and Wood, 2002) and reduce random error and perceptual differences (Mount, 1984; Atwater and Yammarino, 1992; Denison et al., 1995; Hooijberg, 1996; Shipper and Davy, 2002). Due to the little work experience of members of the sample, raters from both the personal (family members, friends) and professional (fellow students, colleagues, superiors, coaches) environments were involved. Each student provided a list of people who knew them well and had seen them in action, coming from different personal and professional environments. The raters were invited through a digital platform and asked to assess the student on the ESCI-U scale.

### Statistical Analyses

To test our hypotheses, we used a Partial Least Square-Path Modeling (PLS-PM). PLS–PM was adopted because it does not make assumptions on data distribution (Fornell and Bookstein, 1982) and can process non-continuous variables (Fornell and Bookstein, 1982; Haenlein and Kaplan, 2004). Moreover, it is a prediction-oriented variance-based approach that is considered to be preferable for exploratory analysis (Fornell and Bookstein, 1982; Hair et al., 2012; Henseler et al., 2014).

In the structural model, we considered as independent variables the student's participation in extracurricular activities and as dependent variables the five groups of social and emotional competencies of the ESCI-U. In the measurement model, the five competency groups were considered as latent variables, as they could not be measured directly. The competencies inside each group were considered as manifest variables. We conducted convergent and discriminant validity analysis to assess the quality of the measurement model. Three control variables were included in the model: field of study (dummy variable business and economics/scientific and humanistic/linguistic study course), Bachelor's degree final grade, and gender.

## Results

Our analysis was carried out in three steps. First, we performed an exploratory data analysis. Correlations between the main variables are presented in Table 2. Frequency of participation in extracurricular activities are summarized in Table 3.

**Table 2:** Correlations between main variables.

		**1**.	**2**.	**3**.	**4**.	**5**.	**6**.	**7**.	**8**.	**9**.	**10**.	**11**.	**12**.	**13**.	**14**.	**15**.	**16**.	**17**.	**18**.	**19**.	**20**.	**21**.	**22**.
1.	Field of study																						
2.	Degree grade	0.10																					
3.	Volunteering	0.13	0.08																				
4.	Cultural	0.28^*^	0.11	0.20^*^																			
5.	Abroad	0.20^*^	0.10	0.14	0.12																		
6.	Sport	0.11	0.12	0.14	0.14	0.16																	
7.	Emotional self-awareness	0.64	−0.02	0.68	0.59	0.66	0.63																
8.	Achievement orientation	0.56	−0.05	0.56	0.48	0.60	0.64^*^	0.50^*^															
9.	Adaptability	0.64^*^	−0.11	0.55	0.60	0.56	0.57	0.42^*^	0.67^*^														
10.	Self-control	0.58	−0.09	0.64	0.60	0.63	0.63	0.36^*^	0.54^*^	0.57^*^													
11.	Positive outlook	0.63	−0.05	0.58	0.55	0.63	0.65^*^	0.40^*^	0.58^*^	0.61^*^	0.60^*^												
12.	Consciousness	0.50	−0.06	0.52	0.52	0.50	0.51	0.35^*^	0.64^*^	0.48^*^	0.34^*^	0.20^*^											
13.	Empathy	0.60	−0.04	0.61	0.64	0.62	0.60	0.53^*^	0.49^*^	0.53^*^	0.61^*^	0.48^*^	0.40^*^										
14.	Organizational awareness	0.51	−0.10	0.47	0.49	0.45	0.52	0.46^*^	0.58^*^	0.62^*^	0.50^*^	0.39^*^	0.48^*^	0.57^*^									
15.	Service orientation	0.48	−0.03	0.53	0.46	0.44	0.49	0.52^*^	0.37^*^	0.49^*^	0.48^*^	0.46^*^	0.39^*^	0.66^*^	0.42^*^								
16.	Conflict management	0.56	−0.07	0.60	0.63^*^	0.53	0.53	0.57^*^	0.54^*^	0.62^*^	0.63^*^	0.63^*^	0.39^*^	0.65^*^	0.57^*^	0.65^*^							
17.	Developing others	0.60	0.00	0.55	0.59	0.64	0.64	0.54^*^	0.54^*^	0.56^*^	0.49^*^	0.55^*^	0.39^*^	0.62^*^	0.53^*^	0.70^*^	0.71^*^						
18.	Influence	0.53	−0.04	0.48	0.42	0.47	0.49	0.61^*^	0.59^*^	0.62^*^	0.48^*^	0.54^*^	0.34^*^	0.55^*^	0.61^*^	0.51^*^	0.70^*^	0.67^*^					
19.	Inspirational leadership	0.60	0.00	0.66	0.60	0.60	0.62	0.54^*^	0.58^*^	0.62^*^	0.47^*^	0.64^*^	0.34^*^	0.51^*^	0.50^*^	0.60^*^	0.69^*^	0.79^*^	0.72^*^				
20.	Team working	0.63	−0.03	0.57	0.60	0.61	0.59	0.48^*^	0.57^*^	0.66^*^	0.47^*^	0.51^*^	0.49^*^	0.59^*^	0.58^*^	0.60^*^	0.66^*^	0.65^*^	0.59^*^	0.70^*^			
21.	Change agent	0.50	−0.02	0.55	0.41	0.53	0.47	0.49^*^	0.64^*^	0.68^*^	0.43^*^	0.51^*^	0.43^*^	0.43^*^	0.55^*^	0.48^*^	0.61^*^	0.65^*^	0.67^*^	0.73^*^	0.60^*^		
22.	System thinking	0.47	−0.03	0.51	0.47	0.46	0.48	0.42^*^	0.53^*^	0.55^*^	0.45^*^	0.42^*^	0.45^*^	0.43^*^	0.56^*^	0.40^*^	0.51^*^	0.58^*^	0.59^*^	0.53^*^	0.52^*^	0.55^*^	
23.	Pattern recognition	0.41	−0.02	0.44	0.46	0.39	0.44	0.44^*^	0.52^*^	0.450^*^	0.42^*^	0.37^*^	0.37^*^	0.47^*^	0.59^*^	0.38^*^	0.52^*^	0.62^*^	0.61^*^	0.51^*^	0.41^*^	0.57^*^	0.57^*^

***p < 0.001, **p < 0.05,

* *p < 0.1*.

**Table 3:** Frequency table of extracurricular activities.

**a)**	**Volunteering**	**Cultural**	**Sport**
Less than once a month	63	54	9
From 1 to 2 times a month	34	33	174
Once a week	28	51	48
Twice or more times a week	18	19	93
Total (sample %)	143 (44.1%)	157 (48.5%)	324 (100%)
**b)**	**Abroad**		
From 1 to 3 months	26		
From 4 to 6 months	68		
From 7 months to 1 year	22		
More than 1 year	8		
Total (sample %)	124 (42.3%)		

Second, we assessed the quality of the measurement model. Convergent validity was assessed through the average variance extracted (AVE). All competency groups showed an AVE larger than the commonly accepted threshold of 0.5 (Hair et al., 2017), indicating that the corresponding latent variable explains more than half the variance in the given indicators (Table 4). To address discriminant validity, we used HTMT (Henseler et al., 2015), which is considered a superior measure compared to the Fornell–Larcker criterion (Latan and Noonan, 2017; Benitez et al., 2020). The HTMT value is required to be <0.90 (Henseler et al., 2015). Results for all the competency groups met this rule of thumb (Table 4). Moreover, we determined discriminant validity by assessing the Maximum Shared Variance (MSV) and the Average Shared Squared Variance (ASV), both of which were found to be lower than the AVE for all constructs (Hair et al., 2010) (Table 4). Reliability was assessed using Cronbach's alpha and Dillon–Goldstein rho. Table 5 summarizes the results, which revealed Cronbach's alpha to be >0.7, and a Dillon–Goldstein rho of >0.87 for all competency groups. Reliability assessment on individual competency scales confirmed the reliability of sub-scales, showing s Cronbach's alpha of >0.7, and a Dillon–Goldstein rho of >0.83. The factor loading estimates from our example are presented in Table 5. All factor loadings, except self-control (0.57) and positive outlook (0.59) in the self-management group, were >0.7, according to the thresholds proposed by Hu and Bentler (2009).

**Table 4:** Convergent and discriminant validity.

**Competency group**	**MSV**	**ASV**	**AVE**	**HTMT and correlations**			
				**Self-management**	**Social awareness**	**Relationship management**	**Cognitive**
Self-awareness			1.000	**0.542**	**0.590**	**0.648**	**0.518**
Self-management	0.503	0.340	0.742	0.618	**0.746**	**0.788**	**0.619**
Social awareness	0.569	0.466	0.685	0.697	0.792	**0.762**	**0.552**
Relationship management	0.655	0.501	0.794	0.730	0.840	0.852	**0.718**
Cognitive	0.438	0.438	0.897	0.646	0.747	0.723	0.793

*Below the diagonal elements are the HTMT values. Above the diagonal elements (in bold) are the correlations between the latent constructs*.

**Table 5:** Measurement model.

	**Factor loadings**					**Cronbach's α**		**DG Rho**	
	**Self-awareness**	**Self-management**	**Social awareness**	**Relationship management**	**Cognitive**				
Emotional self-awareness	1.00					0.87	0.87	0.91	0.87
Achievement orientation		0.89				0.90	0.85	0.93	0.89
Adaptability		0.87				0.90		0.92	
Self-control		0.59				0.94		0.95	
Positive outlook		0.57				0.92		0.94	
Consciousness		0.80				0.87		0.93	
Empathy			0.90			0.92	0.79	0.94	0.88
Organizational awareness			0.83			0.84		0.89	
Service orientation			0.77			0.85		0.91	
Conflict management				0.82		0.80	0.93	0.87	0.94
Developing others				0.88		0.85		0.90	
Influence				0.85		0.82		0.88	
Inspirational leadership				0.92		0.92		0.94	
Team working				0.80		0.91		0.94	
Change agent				0.85		0.82		0.89	
System thinking					0.77	0.75	0.73	0.87	0.88
Pattern recognition					0.96	0.79		0.86	

After confirming the appropriateness of the measurement model, as a third step we analyzed the structural part of the model provided by five regressions. The path coefficient interpretation in PLS-PM is equal to the standardization of regression coefficients (Latan, 2018). The results are summarized in Table 6.

**Table 6:** Structural model.

	**Coeff**.	**SE**	***p*-value**	**Supported**
H1: volunteering—Self-awareness	−0.03	0.060	0.612	No
H1: volunteering—Social awareness	0.05	0.060	0.382	No
H2: cultural—Relationship management	0.14	0.059	0.017^**^	Yes
H2: cultural—Cognitive	0.10	0.060	0.082^*^	Yes^+^
H3: abroad—Self-management	0.16	0.056	0.004^**^	Yes
H3: abroad—Social awareness	0.10	0.057	0.074^*^	Yes^+^
H3: abroad—Relationship management	0.15	0.057	0.010^**^	Yes
H3: abroad—Cognitive	0.16	0.058	0.008^**^	Yes
H4: sport—Self-management	0.13	0.057	0.026^**^	Yes
H4: sport—Relationship management	0.00	0.058	0.981	No
GOF	0.213			

***p < 0.001;

** *p < 0.05*;

* *p < 0.1*;

+ *significant at 10% level*.

Hypothesis 1 on volunteering was not supported (self-awareness β = −0.03, *p* > 0.1; social awareness β = 0.05, *p* > 0.1). As for H2, we found support for the relationship between the participation in cultural activities and the relationship management competency group (β = 0.14, *p* < 0.05). Findings seemed also to support the positive influence of cultural activities on the cognitive group, with results significant at the *p* = 10% level (β = 0.10, *p* < 0.1). As for experiences abroad (H3), we predicted a positive relationship with self-management (β = 0.16, *p* < 0.05), social awareness (β = 0.10, *p* < 0.1), relationship management (β = 0.15, *p* < 0.05), and cognitive competencies (β = 0.16, *p* < 0.05). The relationship with social awareness was tentatively supported, whereas the positive relationship between experiences abroad and self-management, relationship management, and cognitive competencies found evidence in our empirical analysis. Concerning Hypothesis 4, partial support was found. According to our results, participation in sport activities is positively related to higher self-management competencies (β = 0.13, *p* < 0.05), but not significantly related to the relationship management competencies (β = 0.00, *p* > 0.1). The category of sport activity used in the model included both individual and team sports; nevertheless, when conducting the analysis by separating the two types of sport activities no significant difference was found.

## Discussion

This study adds to the literature by investigating the role of extracurricular activities as a relevant dimension of young people's learning experience. These activities “are generally voluntary, have regular and scheduled meetings, maintain developmentally based expectations and rules for participants in the activity setting (and sometimes beyond it), involve several participants, offer supervision and guidance from adults, and are organized around developing particular skills and achieving goals” (Mahoney et al., 2005, p. 4). A growing body of studies has reported the benefits of a consistent participation in ECAs with specific regard to academic (grades, school engagement, educational aspirations), psychological (higher self-esteem and lower rates of depression), and social (lower dropout rates, civic engagement) outcomes (see for instance Fredricks and Eccles, 2006).

More recently, career development studies have highlighted the importance of encouraging such activities in high and higher education, since through the engagement in ECAs students have the opportunity to explore their interests and aptitudes and consequently to shape their career identity (You, 2020; Kanar and Bouckenooghe, 2021) and develop skills linked to their future professional careers (Khasanzyanova, 2017). Indeed, according to Mahoney et al. (2003), the three fundamental elements that characterize ECAs, namely voluntary participation, structure and challenge, are salient in promoting individual competency development. As a matter of fact, is has become a common practice for recruiters to infer job-related behavioral competencies in young applicants, who may lack significant work experience, based on their extracurricular activities (Clark et al., 2015). Nevertheless, very little is found in the literature concerning whether participating in ECAs cultivate ESCs and which activities favor which competencies. By applying the lenses of experiential learning theory to the understanding of competency development in ECAs, the current study addresses the following gaps in the literature: First, in prior studies, different extracurricular activities and several behavioral competencies were aggregated into single measures with the consequent loss of information of the influence of single types of activities on specific types of competencies (see for instance Feraco et al., 2021). Second, the few studies that have analyzed empirically the impact of extracurricular activities on behavioral competencies have primarily considered interpersonal skills, neglecting the other components of ESCs (see for instance Rubin et al., 2002). Alternatively, some prior studies have focused on the influence of just one specific extracurricular activity on individual skills (see for instance Chanda and Levitin, 2013), or have adopted a narrative and descriptive approach to understanding this relationship (see for instance Kanar and Bouckenooghe, 2021). Consequently, findings from these studies cannot not be compared, due to the different methodologies and measurement techniques employed. Addressing these gaps, the present study has contributed to disentangle and examine empirically the relationship between a broad range of ECAs (cultural, volunteering, sport and experiences abroad) and different groups of behavioral competencies (self-awareness, self-management, social awareness, social management and cognitive competencies). Specifically, results show a significant relationship between cultural activities and relationship management abilities. The engagement in cultural activities provides opportunities for discussion and interaction between people and students have to engage with members of a band or orchestra or theater company, which provides an opportunity to spur their abilities in managing social relationships. A positive relationship seems also to characterize cultural activities and cognitive competencies. Activities related to fine arts usually imply the involvement of students in moments and exercises of interpretation in which they have to stress their reasoning. Our findings are consistent with studies claiming that art learning spurs the ability to bridge seemingly disconnected information and experiences (Snyder et al., 2009).

Experiences abroad were found to predict better relationship management, and to a lesser extent social awareness, competencies. Our findings seem to be in agreement with recent studies (Genkova et al., 2021) that show that experiences studying abroad are more strongly associated with social management than with social awareness competencies. Being exposed to a different culture helps the student practice how to deal with diversity and how to manage relationships with people from different backgrounds. Specifically, current research suggests that experiences abroad foster networking development (Prieto-Arranz et al., 2021) and engaging with diversity (Gearing et al., 2020).

Our findings also support the positive association between experiences abroad and self-management and cognitive competencies.

Practicing sport activities is shown to influence positively self-management competencies. Discipline, deeply embedded in sport activities, forces students to deal with the necessity of controlling their positive and negative emotions, directing their own energy and ambitions, and finding new ways to improve. However, no positive influence was found between sport activities and relationship management competencies. This is consistent with Rubin et al. (2002), who found membership of sports teams not to be associated with increased interpersonal skills.

Volunteering was also found not to be related to higher ESCs. The explanation could be that while sports, cultural activities and experiences abroad are often characterized by the expectation of achievement, which thus demand higher self-regulation, social control and adjustment, in volunteering activities the mere presence or contribution in itself is appreciated, and expectation of outcomes is often lacking. Indeed, some prior studies (see, for example, Holdsworth and Quinn, 2010) started to challenge the win-win view of volunteering activity, claiming that the benefits of student volunteering are assumed rather than proven.

Findings from this study provide preliminary evidence that not all ECAs impact ESCs with the same level of intensity. The characteristics of the activities performed by students, the types of challenges in which they are involved and the kind of support and feedback they receive during their experience might contribute to differentiating the learning outcomes. This study also offers counterintuitive results, showing a lack of association between some ECAs and a graduate's competency portfolio, which is instead usually taken for granted in job applicant assessments. These associations require further exploration to better define the concrete learning impact of those activities in terms of competency development. This can be attained through the development of new metrics and classifications that might better represent the complex nature of these activities and capture the different qualities of an experience.

## Implications

A big challenge in the development of ESCs is the necessary involvement of the student in contexts in which he/she can practice new behavioral repertoires; this is achieved through the application of experiential methods (Hoover et al., 2010). Previous studies have focused on experiential learning activities assigned during courses (Vaatstra and de Vries, 2007; Paladino, 2009; Landau and Meirovich, 2011), or have devoted their attention to the impact of specific programs on competency development (McEnrue et al., 2009; Sheehan et al., 2009) but have not taken into account the role of personal extracurricular experiences in enhancing ESCs. In this regard, our study offers theoretical, methodological and practical implications to the debate of behavioral competency development.

From a theoretical perspective, we contribute to the ESC literature by advancing the understanding of the development of ESCs outside the conventional classroom-lecture setting. Specifically, we provided a bridge between the literature on ECAs, which has primarily focused attention on students' academic achievement and well-being, and ESC theory, which has provided evidence of the positive impact of ESCs on individual performance and career development. The frequent claim that ECAs represent a viable space for developing behavioral competencies has received, so far, little empirical investigation. In analyzing this relationship, we have adopted the lenses of experiential learning theory (Kolb, 1984, 2015), which claims that “learning is a process of creating knowledge through synergistic transactions between learners and their environments, and that learners are active drivers of their learning processes” (Trinh et al., 2021, p. 3). We assumed that a recursive process of experiencing, reflecting, thinking and acting is activated by the fact that individuals demonstrate a voluntary participation in ECAs and that these activities challenge their skills through experiences that bring them out of their comfort zone.

From a methodological point of view, this article suggests the need to adopt 360-degree or multi-rater assessments of ESCs, which allows integrating different observations, defining a more comprehensive assessment and avoiding self-perception bias.

Moreover, this study provides implications for designing and complementing ESC development programs in higher education. Universities are recognizing their responsibility toward their students in terms of career development and employment and are striving to provide them with the extracurricular learning experiences that might equip them with those behavioral competencies highly demanded in the labor market (You, 2020). Our claim is that higher education should promote these ECAs inside or outside the university campus and support students through a critical reflection on how to acquire new tools to practice specific ESCs in different situations. As development of behavioral competences requires practice, and frequently the bigger challenge is to find a context to practice, and the energy to persist in the practice of, new behaviors (Boyatzis and McKee, 2005), it is important to encourage students to participate in activities that could foster the development of their ESCs and make them aware of how to exploit this opportunity for their personal growth. Moreover, ECAs could become a stimulus for discussion in class, giving students the opportunity to reflect on their adopted behaviors and the related outcomes.

University career services, in providing guidance to students entering the labor market, could strengthen the potential value of ECAs by helping students to analyze their ECAs in career terms. For instance, students can be supported in making ECAs more visible in their curriculum vitae and in explaining explicitly in which way the specific activity could be associated to some ESCs.

This study offers an additional interest in terms of managerial implications, as inferring ESCs from the presence of ECAs in a student's curriculum vitae is a well-known common practice, which is nevertheless based more on common feeling than on scientific inquiry. This study contributes to create a debate from which to develop clearer and more scientifically based recruiting processes. Employers who use ECAs to gauge the quality of or differentiate between candidates (Cole et al., 2007) can benefit from empirical analysis that disentangles the predictive role of different types of ECA. This can enhance the awareness that ESCs are not equally associated with all types of ECA, as well as a better understanding of what ESCs they might expect in individuals with different extracurricular experiences.

## Limitations and Future Research

Some limitations and recommended directions for future research can be identified. Firstly, limitations in terms of size, gender and geographical composition of the sample may constitute a threat to the external validity of this study. Secondly, we would like to address the issue of causation often raised in this type of study. Our assumption is that the choice of practicing a certain ECA may depend not necessarily on the fact that the person feels in tune with the activity due to ESCs he/she has already acquired but on many other contingencies. In this regard, prior studies identified children's achievement-related motivation, family demographic factors and parenting processes as relevant predictors of ECA participation (Fredricks and Eccles, 2006). According to previous studies, ESCs are personal characteristics that are not innate from birth but are learned and developed during life (Fineman, 1997; Goleman, 1998), especially during the phase of development of young individuals that are the target group of this study. Our study, which takes into account the intensity with which the ECA is performed, is coherent with the theories of ESC learning, according to which the more a behavior is practiced the more it becomes an automatic response of the brain; therefore, it translates into a common behavior and a persistent competency (Goleman et al., 2002; Boyatzis, 2008a). This assumption is consistent with the results of the longitudinal study conduct by Mahoney et al. (2003), who showed that participation in ECAs in both early and middle adolescence is associated with increased interpersonal competence over time. In order to address this issue in greater detail, we suggest the use of a quasi-experimental design with pre- and post-tests on the level of ESCs and an intervention concerning the experience of an ECA. The manipulation of participation and the features of the activity setting allows seeing which are the critical aspects of an ECA that nurture changes in individual behaviors. Furthermore, although this study looked at intensity, which is one of the most commonly assessed dimensions in extracurricular studies (Bohnert et al., 2010), it did not take into account the length and quality of the students' involvement. A more sophisticated measure of participation in ECAs, such as duration/consistency and engagement (Bohnert et al., 2010), should be considered in future research in order to take into account those factors that might moderate the relationship between activity participation and ESC development. Also, the breadth of the participation in ECAs (Bohnert et al., 2010) could be considered in future research in order to understand whether an activity that is carried out jointly with other activities has a different impact on the competency profile of an individual. In this regard, complementarities or synergies among ECAs represent a promising line of research.

Moreover, ECAs do not occur in isolation. Contextual-level factors may influence the intensity of the participation and the engagement of the student in each stage of Kolb's experiential learning cycle, affecting the subsequent level of behavioral competency development. For instance, although extracurricular activities naturally and even unconsciously spur young people to be engaged into concrete experience and active experimentation, the other two stages of Kolb's learning cycle (reflective observation and abstract conceptualization) may require a more conscious effort, thus are more likely to occur when stimulated by structured or supervised relationships. Coaches and teachers, who act as sources of support and feedback, may promote the process of making sense of events and understanding the linkages between them. Prior studies considered their role paramount in promoting a sustainable behavioral change (Kampa-Kokesch and Anderson, 2001; Boyatzis et al., 2010). Effective coaching helps the individual identify habitual scripts of behavior and understand their outcomes, reveal fresh insights into what drives one's behavior and convert those insights into observable behavior change (Brotman et al., 1998). Stimulating individual learning and change, especially through coaching with compassion rather than coaching with compliance, turns out to be extremely beneficial (Boyatzis et al., 2013). The importance of training teachers and adult supervisors should not be neglected. In fact, the sports psychology literature provides compelling evidence that supervisors and trainers, when not trained to give feedback and emotional support, can have a negative effect on students' development (Eccles et al., 2003). Thus, future research should consider those contextual mechanisms that might moderate the activation of the overall four-stage learning cycle and the effectiveness of personal development. Finally, as ECAs can be conceived and practiced in different ways in different cultures, we suggest that future research should replicate this study in other cultural settings.

## Data Availability Statement

The raw data supporting the conclusions of this article will be made available by the authors, without undue reservation.

## Ethics Statement

Ethical review and approval was not required for the study on human participants in accordance with the local legislation and institutional requirements. The patients/participants provided their written informed consent to participate in this study.

## Author Contributions

LC and SB contributed to the conception and design of the study and manuscript revision. LC, SB, and FG contributed to the data collection. CP defined the method and performed the data analysis. LC wrote the first draft of the manuscript. All authors contributed to the article and approved the submitted version.

## Conflict of Interest

The authors declare that the research was conducted in the absence of any commercial or financial relationships that could be construed as a potential conflict of interest.

## Publisher's Note

All claims expressed in this article are solely those of the authors and do not necessarily represent those of their affiliated organizations, or those of the publisher, the editors and the reviewers. Any product that may be evaluated in this article, or claim that may be made by its manufacturer, is not guaranteed or endorsed by the publisher.
